# CXCL12-PLGA/Pluronic Nanoparticle Internalization Abrogates CXCR4-Mediated Cell Migration

**DOI:** 10.3390/nano10112304

**Published:** 2020-11-20

**Authors:** Anissa Pisani, Roberto Donno, Arianna Gennari, Giulia Cibecchini, Federico Catalano, Roberto Marotta, Pier Paolo Pompa, Nicola Tirelli, Giuseppe Bardi

**Affiliations:** 1 Nanobiointeractions & Nanodiagnostics, Istituto Italiano di Tecnologia, Via Morego 30, 16163 Genova, Italy; anissa.pisani@iit.it (A.P.); giulia.cibecchini@iit.it (G.C.); pierpaolo.pompa@iit.it (P.P.P.); 2 Department of Chemistry and Industrial Chemistry, University of Genova, Via Dodecaneso 31, 16146 Genova, Italy; 3 Laboratory of Polymers and Biomaterials, Istituto Italiano di Tecnologia, 16163 Genova, Italy; roberto.donno@iit.it (R.D.); arianna.gennari@iit.it (A.G.); 4 Electron Microscopy Laboratory, Istituto Italiano di Tecnologia, Via Morego 30, 16163 Genova, Italy; federico.catalano@iit.it (F.C.); roberto.marotta@iit.it (R.M.); 5 Division of Pharmacy and Optometry, School of Health Sciences, University of Manchester, Oxford Road, Manchester M13 9PT, UK

**Keywords:** nanoparticles, chemokines, chemokine-receptor, cell-migration, immune cells, cancer

## Abstract

Chemokine-induced chemotaxis mediates physiological and pathological immune cell trafficking, as well as several processes involving cell migration. Among them, the role of CXCL12/CXCR4 signaling in cancer and metastasis is well known, and CXCR4 has been often targeted with small molecule-antagonists or short CXCL12-derived peptides to limit the pathological processes of cell migration and invasion. To reduce CXCR4-mediated chemotaxis, we adopted a different approach. We manufactured poly(lactic acid-co-glycolic acid) (PLGA)/Pluronic F127 nanoparticles through microfluidics-assisted nanoprecipitation and functionalized them with streptavidin to docking a biotinylated CXCL12 to be exposed on the nanoparticle surface. Our results show that CXCL12-decorated nanoparticles are non-toxic and do not induce inflammatory cytokine release in THP-1 monocytes cultured in fetal bovine and human serum-supplemented media. The cell internalization of our chemokine receptor-targeting particles increases in accordance with CXCR4 expression in FBS/medium. We demonstrated that CXCL12-decorated nanoparticles do not induce cell migration on their own, but their pre-incubation with THP-1 significantly decreases CXCR4^+^-cell migration, thereby antagonizing the chemotactic action of CXCL12. The use of biodegradable and immune-compatible chemokine-mimetic nanoparticles to reduce cell migration opens the way to novel antagonists with potential application in cancer treatments and inflammation.

## 1. Introduction

Chemoattractant cytokines, broadly known as chemokines, are ~10 kDa proteins whose major task is the induction of cell migration [1]. Beyond their key role in immune cell homing and inflammatory trafficking [2,3], the involvement of the chemokine–chemokine receptor system in cancer is becoming more and more clear [4]. In particular, the upregulation of CXCL12 (formerly known as Stromal derived factor 1, SDF-1) and of its cognate receptor CXCR4 significantly correlates with metastasis and poor cancer prognosis, as reported in a comprehensive review article by Wang and colleagues [5]. Preclinical and clinical observations of high CXCR4 and CXCL12 expression levels in different tissues affected by cancer cell invasion highlight their importance as potential targets for delivery strategies with advanced therapies [6,7,8,9,10,11,12]. Ligand-mediated drug delivery exploiting the overexpression of a defined membrane protein is one of the major strategies for specific cell targeting.

CXCR4, as all the chemokine receptors, belongs to the 7-helix trans membrane G protein-coupled receptors (7TM GPCRs) superfamily. Receptor interaction with CXCL12 was elucidated several years ago [13]. CXCL12 binding to CXCR4 is mediated by the chemokine loop between the cysteines and the N-terminal region entering the groove at the top of the receptor helices. The deletion of the first amino acids at the N-terminus of the chemokine creates peptides with a reduced binding affinity and an insufficient ability to induce intracellular signaling (e.g., Ca^2+^ release from the intracellular stores).

The CXCL12/CXCR4 axis plays crucial roles in cell homeostasis and the development of organs coordinating the movement of different cells during the embryogenesis and regeneration of adult cells [14]. In addition to immune cell circulation from and to hematopoietic tissues, CXCR4/CXCL12 interaction triggers different biological events, like natural killer cell development [15], Igκ-chain recombination during B lymphocyte differentiation [16], and B cells and granulocyte retention within the bone marrow [17], among several others. CXCR4 is also expressed on the microglia, astrocytes and neurons in the brain [18]. In the nervous system, CXCL12 behaves as a neuromodulator in pathological conditions, such as pain [19], ischemic stroke [20] or cross-talking with opioid receptors [21].

Unfortunately, viruses can exploit CXCR4 as an entry point into cells. Incidentally, diverse peptides show binding affinity for some chemokine receptors. For instance, the HIV envelope protein gp120 binds CCR5 and CXCR4 after conformational changes due to the previous interaction with CD4 [22,23]. The contact between the HIV envelope proteins and the chemokine receptors is fundamental for virus entry and infection. A mutation in the CCR5 gene inducing a deletion at the residue 32 can prevent the monocyte-tropic virus’ internalization, and impede infection in the homozygous carriers [24]. The gp120-mediated effects on CXCR4 depend on the presence of the CD4 coreceptor, resulting in agonist or antagonist properties, as well as modifications of CXCL12/CXCR4 signaling [25,26].

In search of drugs to avoid HIV entry, T22 ([Tyr^5,12^, Lys^7^]-polyphemusin II), a synthetic peptide derived from basic peptides isolated from the blood cells of horseshoe crabs, showed interesting agonistic activity in relation to CXCR4 [27]. T22 has been engineered to prepare self-assembled protein-only nanoparticles (NPs) or fusion-protein drug carriers, and was successfully applied in different CXCR4^+^-expressing tumor cells in vitro and in vivo [28,29,30,31,32,33,34,35,36]. The deletion of cancer cells was been obtained by the T22-mediated precise dissemination of anticancer drugs to the primary tumor and metastatic foci. For example, doxorubicin loaded mesoporous SiO_2_ NPs have been driven to B-cell non-Hodgkin’s lymphoma cells by their surface functionalization with an azide-containing T22 analogue peptide [37]. A 5-amino acid-peptide antagonist of CXCR4, called LFC131, has also been used to decorate polymeric NPs loaded with docetaxel [38]. LFC131 conjugation allowed enhanced NP-mediated drug delivery in lung cancer cells overexpressing CXCR4. Short peptides consisting of the N-terminal sequence of CXCL12, or viral chemokines targeting the same receptor, can exhibit high gene-delivery efficiency, increasing the CXCR4^+^ cell uptake of peptide/DNA complexes [39,40].

In a previous work aimed at developing NPs for specific targeting, we covalently bound an entire chemokine on the surface of SiO_2_ NPs [41], whose structure and steric hindrance can limit the opsonization of other proteins. The CXCL5-SiO_2_ NPs’ internalization through the CXCL5 cognate receptor (CXCR2) was increased with regard to the non-chemokine modified particles, even in the presence of serum. Furthermore, we showed that the entire CXCL5 on the SiO_2_ NP’s surface selectively targets high-CXCR2^+^ cells and weakly binds low-CXCR2-expressing cells.

In the present work, we synthesized Pluronic 127 coated-poly(lactic acid-co-glycolic acid) (PLGA) NPs by microfluidics-assisted nanoprecipitation and functionalized their surface with CXCL12 (CXCL12-NPs). Our particles were characterized by static and dynamic light scattering, immuno negative staining and cryo-electron microscopy (cryo-EM). Moreover, their behavior was assessed in vitro. Specifically, CXCR4-expressing human THP-1 monocytic leukemia cells cultured in different sera, namely fetal bovine serum (FBS) and human serum (HS), were chosen as the cell model. NP biocompatibility was examined for potential toxicity and inflammatory cytokine release. CXCL12-NP internalization in THP-1 was comprehensively evaluated by flow cytometry and confocal microscopy in FBS- and HS-supplemented media at different time points, and compared with control NPs without chemokine-surface modification. Finally, we observed the agonistic or antagonistic properties of CXCL12-NPs by chemotaxis assay, pondering cell migration as the main biological function of chemokines and the principal tissue-invading mechanism of metastatic cells overexpressing CXCR4.

## 2. Materials and Methods

### 2.1. Nanoparticle Preparation and Characterization

#### 2.1.1. Materials

Poly(D,L-lactide-co-glycolide) RG502 Resomer (PLGA, acid terminated PLGA-COOH), Pluronic F127, biotin, cysteamine hydrochloride, 2-mercaptoethanol, sodium azide, d6-dimethylsulfoxide (d6-DMSO), and Spectra/Por^®^ dialysis membranes (MWCO 3.5 kDa) were purchased from SigmaAldrich (Merk Life Science, Milan, Italy). HEPES sodium salt and 2 M hydrochloric acid solution were supplied from Alfa Aesar (Thermo Fisher, Kandel, Germany). The 4-(4,6-dimethoxy-1,3,5-triazin-2-yl)-4-methylmorpholinium chloride (DMTMM) was from Fluorochem (Hadfield, UK).

#### 2.1.2. Preparation of Pluronic F127-Biotin (F127-BIO)

Pluronic F127-α,ω-bis(vinyl sulfone) (F127-VS) with 63% molar percentage of OH groups converted into vinyl sulfone moieties was prepared as previously reported [42]. In total, 100 mg of F127-VS (corresponding to 10 µmol of vinyl sulfone groups) were dissolved in 25 mL of 50 mM HEPES buffer at pH 7.4 and sonicated for 10 min to degas. Then, 1.4 mg (12 µmol, 1.2 eq.) of cysteamine hydrochloride was added and the solution was left overnight on an orbital shaker. Afterwards, in a second reaction vessel, 7 mg of biotin (30 µmol, 2.5 eq.) dissolved in 100 µL of DMSO was mixed for 5 min with 12 mg of DMTMM (45 µmol, 3.8 eq.) dissolved in 200 µL of HEPES buffer. DMTMM and biotin were then added to the modified Pluronic. After 24 h a large excess of 2-mercaptoethanol (1.5 mL) was added in order to quench all the remaining vinyl sulfone groups and the reaction was allowed to proceed overnight. The product was finally purified by dialysis against deionized water (MWCO 3.5 kDa) and recovered after freeze drying with 80% mass recovery.

Quantitative conversion of the double bond was confirmed by ^1^H NMR. Specifically, 87% (molar percentage) of the vinyl sulfone groups were biotinylated, while the remaining groups were quenched with 2-mercaptoethanol. ^1^H NMR was recorded on 1 wt.% polymer solutions in d6-dimethylsulfoxide (d6-DMSO) using a Bruker AVANCE III 400 MHz spectrometer equipped with a Broad Band Inverse probe. The residual solvent signal was used as internal reference (ppm).

**Figure 1:**
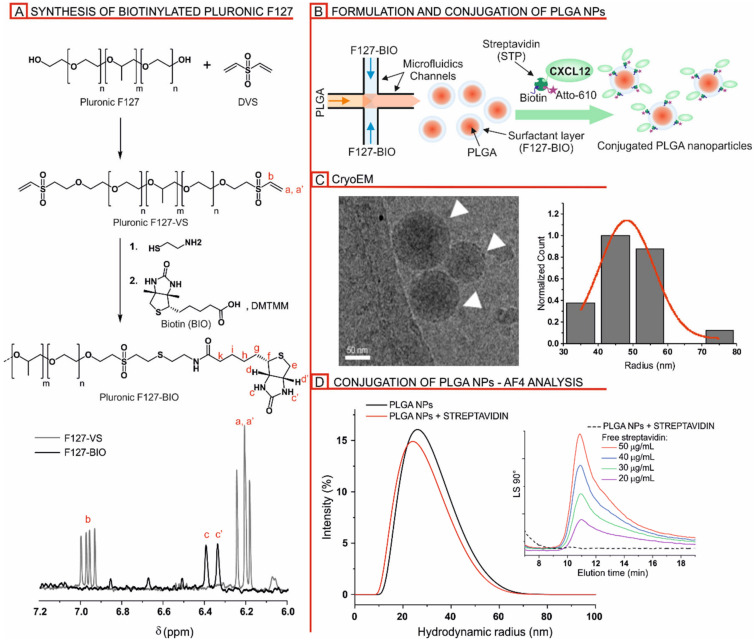
(**A**) Top: Scheme of the synthesis of biotinylated Pluronic F127. Bottom: Over-imposed ^1^H NMR spectra of Pluronic F127-VS (dashed grey line) and Pluronic F127-BIO (solid black line). (**B**) Schematic diagram of the microfluidic-assisted nanoprecipitation of PLGA NPs coated with Pluronic F127-BIO (25% w/w of the total amount of surfactant) and streptavidin-mediated decoration with CXCL12. (**C**) Left: representative cryoEM image of frozen hydrated PLGA NPs (arrowheads). Right: Gaussian curve fitted NP size distribution. (**D**) Hydrodynamic size of PLGA NPs coated with Pluronic F127-BIO (25% w/w of the total amount of surfactant) before and after the addition of 0.5 equivalent of streptavidin compared to the amount of biotin on the F127-BIO. Please note that streptavidin was first reacted with 3 equivalents of biotin in order to quench three of the four available biotin-binding sites, minimizing the possibility of nanoparticle agglomeration. Size distributions were obtained by fractionating the samples by AF4 and using a DLS as the online detector. Inset: AF4 elugrams obtained after injection of free streptavidin at different concentrations and streptavidin first quenched with 3 equivalents of biotin and then mixed with PLGA NPs (25% F127-BIO) in a 1:2 molar ration compared to the biotin on the F127-BIO (dashed line) (final streptavidin concentration: 50 µg/mL).

Pluronic F127-biotin (F127-BIO): d6-DMSO, δ (ppm): 0.81–0.92 (m, H-h), 1.04 (d, *J* = 8 Hz, methyl group in propylene oxide units), 1.15–1.40 (m, H-g), 1.43–1.55 (m, H-i), 2.57–2.65 (m, H-k), 2.80–2.85 (m, H-e), 3.48–3.54 (m, methylene and methine groups in both ethylene and propylene units), 4.12–4.16 (m, H-d, d’), 6.34 (bs, H-c), 6.39 (bs, H-c’). For proton assignment, please refer to Figure 1A.

#### 2.1.3. Nanoparticle Preparation

The automated microfluidic Asia 320 system (Syrris, Royston, UK) was used for all preparations. A 0.015 wt.% surfactant aqueous solution (25% Pluronic F127-VS and 75% Pluronic F127) was mixed with a 0.31 wt.% PLGA acetone solution in an Asia reaction chip (26 μL Micromixer chip, Syrris, Royston, UK). The flow rates were controlled to have an acetone/water flow rate ratio of 0.2 and a total flow of 2 mL/min.

The collected nanoparticle (NP) suspension was left at 30 °C for 12 h under stirring in order to evaporate the acetone. MilliQ water was then added in amounts equal to the volume loss, in order to maintain the initial nanoparticle concentration (i.e., 0.052 wt.%). The NPs suspension was then sterilized by filtering it with 0.22 µm cellulose acetate filters.

#### 2.1.4. Nanoparticle Decoration

The streptavidin-mediated decoration of PLGA NPs involved two steps: firstly, streptavidin was mixed with 3 equivalents of biotin-containing molecules in order to occupy three of the four available binding sites; secondly, the aforementioned mixture was added to the biotinylated NPs with a 2:1 molar ratio between the biotin bound on the NPs and the streptavidin. Specifically:

(a) Control NPs—10 μL of a 100 μM solution of Atto 610-biotin (Sigma-Aldrich, Saint Louis, MO, USA) in DMSO (equivalent to 1 nmol) was mixed with 290 µL of 7 μM solution of biotin in MilliQ water (equivalent to 2 nmol), followed by 50 μL of streptavidin reconstituted at 1 mg/mL, corresponding to 1 nmol of protein units. The procedure was allowed to proceed for 15 min. The mixture was then added to 650 µL of the 0.052 wt.% nanoparticle dispersion (biotin content = 2 nmol), vortexed for 10 s and allowed to rest for 15 min.
(b) CXCL12-decorated NPs—10 μL of a 100 μM solution of Atto 610-biotin in DMSO (equivalent to 1 nmol) was mixed with 280 µL of 7 μM solution of biotin in MilliQ water (equivalent to 1.96 nmol), and 10 μL of a 10 μM solution of biotinylated human CXCL12 in MilliQ water (equivalent to 0.1 nmol) (Chemotactics, San Diego, CA, USA), followed by 50 μL of streptavidin (Prospec, Rehovot, Israel) reconstituted at 1 mg/mL, corresponding to 1 nmol of protein units. The procedure was allowed to proceed for 15 min. The mixture was then added to 650 µL of the 0.052 wt.% nanoparticle dispersion (biotin content = 2 nmol), vortexed for 10 s and allowed to rest for 15 min.

#### 2.1.5. Cryogenic Transmission Electron Microscopy (Cryo-EM)

In total, 3µL measures of the NP suspension (directly from preparation after evaporation of acetone) were deposited on glow discharged holey carbon grids (200 mesh, Quantifoil, Ted Pella) followed by vitrification using a FEI Vibrot Mark IV (FEI Company, Eindhoven, Netherlands) (1.5 s blotting, 4 °C, 90% relative humidity) in liquid ethane cooled at liquid nitrogen temperature. Imaging was performed at cryogenic temperature (i.e., below −170 °C) using a Tecnai G2 F20 cryo transmission electron microscope equipped with a Schottky field emission electron (FEG) source operating at 200 kV, a US1000 2k × 2k Gatan charge-coupled device (CCD) camera (GATAN, Pleasanton, USA) and a FEI Retractable Cryo Box (FEI Company, Eindhoven, Netherlands). The projection images were acquired with a total electron dose of 10 electrons/A^2^ and a final pixel size of 3.6 Å.

#### 2.1.6. Asymmetric Flow Field-Flow Fractionation (AF4)

PLGA NPs coated with F127/F127-BIO (75:25 weight ratio) were prepared with a microfluidic chip as described above. The NPs were then decorated with streptavidin previously pre-saturated with 3 equivalents of biotin according to the protocol reported above. Samples were then analyzed by asymmetric flow field-flow fractionation (AF4) in water.

The AF4 system AF2000 TM (Postnova Analytics, Landsberg, Germany) was coupled online to UV/VIS at 280 nm (S3210, Laserchrom, Rochester, UK), PN3609 multi-angle light scattering (MALS) (Postnova Analytics, Landsberg, Germany), PN3150 refractive index (Postnova Analytics, Landsberg, Germany) and Mobius dynamic light scattering (DLS) (Wyatt, Santa Barbara, California) detectors in the given order. The AF4 channel was equipped with a 350 µm spacer and a 10 kDa MWCO membrane of regenerated cellulose as the accumulation wall. A solution of 0.02% (w/v) NaN_3_ was filtered through a 0.1 µm filter and used as eluent.

Nanoparticle analysis: in a typical experiment, the detector flow rate was set at 0.5 mL/min and 100 μL measures of the samples were injected over 5 min at 0.2 mL/min with a crossflow of 1.0 mL/min and a focusing flow of 1.3 mL/min (focusing step). For the elution step, the crossflow was kept constant at 1.0 mL/min for 0.2 min and then exponentially (exponent = 0.20) decreased to 0.1 mL/min over 40 min, and subsequently kept at 0.1 mL/min for an additional 20 min, followed by a 2 min rinse step (i.e., crossflow at 0 mL/min and purge valve on). The data collected by the MALS and RI detectors were analyzed on AF2000 software (Postnova Analytics) and fitted with a sphere model to obtain the radius of gyration distribution. The hydrodynamic size was measured in flow using Dynamics software (Wyatt).

Analysis of residual free streptavidin: the detector flow rate was set at 0.5 mL/min and 50 μL measures of the samples were injected over 5 min at 0.2 mL/min with a crossflow of 2.5 mL/min and focusing flow of 2.8 mL/min (focusing step). For the elution step, the crossflow was kept constant at 2.5 mL/min for 15 min and then linearly decreased to 0 mL/min over 5 min.

#### 2.1.7. Dynamic Light Scattering (DLS)

Hydrodynamic size, size polydispersity (PDI) and Z-potential were measured on control and CXCL12-decorated NPs in batch mode (DLS not used as AF4 detector) at a temperature of 25 °C using a Möbiuz instrument (Wyatt Technology, Santa Barbara, CA, USA) equipped with a laser at 532 nm and a scattering angle of 163.5°. the DLS acquisition time and number were set at 1 s and 5, respectively. Correlation functions were analyzed by Dynals algorithm. The electrophoretic mobility was measured for 5 s using a voltage amplitude of 3 V, 10 Hz electric field frequency, and converted into Z-potential using Smoluchovski equation.

#### 2.1.8. Negative Staining EM Immunolabeling

Here, 5 μL drops of samples were deposited on pure carbon 300 mesh copper grids. After 2 washing steps on 50 μL wash buffer (0.1% Bovine Serum Albumin (BSA) (Miltenyi Biotec, Bergisch Gladbach, Germany) in 1× Phosphate Buffered Saline (PBS) (Sigma-Aldrich, Saint Louis, MO, USA)) the grids were incubated in 50 μL drops of Rabbit Anti-Human CXCL12 (PeproTech, Rocky Hill, NJ, USA) in buffer A (1%BSA in PBS) in a wet chamber for 1 h at RT. After incubation the grids were washed (5 washes) in washing buffer and then incubated for 30 min on a 20 μL drop of 1:40 dilution of antirabbit IgG-gold conjugate (6 nm particle size) (Aurion CliniSciences, Nanterre, France) in wash buffer. After subsequent washes on 5 drops of wash buffer and 5 washes on water drops, the grids were negative stained with uranyl acetate (Polysciences, Inc. 400 Valley Road Warrington, PA, USA) (1% in water) for 60 s. The resulting samples, called CXCL12 and negative-CTRL, were then analyzed by transmission electron microscopy (TEM). A JEM-1011 (JEOL, Tokyo, JAPAN) transmission electron microscope was used, with the thermionic source (W filament) operating at an acceleration voltage of 100 kV. The TEM image detector was a Gatan Orius SC1000 series CCD camera (4008 × 2672 active pixels) (GATAN, Pleasanton, USA), fiber optically coupled to high-resolution phosphor scintillator. In total, 30 images were acquired and Au-labeled NPs were expressed as a percentage relative to the total amount of NPs counted per sample (Negative-CTRL and CXCL12).

### 2.2. Cell Culture

THP-1 cells (ATCC, Manassas, VA, USA) were grown in RPMI-1640 (Thermo Fisher Scientific, Waltham, MA, USA) supplemented with 10% FBS or 5% HS (Thermo Fisher Scientific, Waltham, MA, USA), 1% Penicillin-Streptomycin (Sigma-Aldrich, Saint Louis, MO, USA), and 0.05 mM 2-mercaptoethanol (Thermo Fisher Scientific, Waltham, MA, USA) in a 5% CO_2_ humidified atmosphere at 37 °C.

### 2.3. Toxicity Assay

Cell metabolic activity was determined using a WST-8 (2-(2-methoxy-4-nitrophenyl)-3-(4-nitrophenyl)-5-(2,4-disulfophenyl)-2H-tetrazolium, monosodium salt) assay (Sigma-Aldrich, Saint Louis, MO, USA) following the manufacturer’s instructions. Briefly, after 24 h incubation with 32.5 µg/mL of CXCL12-NPs or control NPs, cells were washed twice, resuspended in complete culture medium (supplemented with 10% FBS or 5% HS) and seeded at a density of 5 × 10^4^ cells/100 µL in 96-well plates (Corning, Corning, NY, USA). In total, 10 µL measures of Cell Counting Reagent WST-8 were added to each well and the plates were incubated in a 5% CO_2_ humidified atmosphere at 37 °C for 3 h. The orange WST-8 formazan product was measured on a Sparck multimode microplate reader (Tecan, Männedorf, Switzerland) at a wavelength of 460 nm, and 10% DMSO for 24 h was used as positive control.

### 2.4. Cytokine Release

THP-1 cultured in 5% HS/medium was collected (2 × 10^5^ cells/mL) and resuspended in 0.5% HS/medium at 37 °C. After 30 min at rest, the cells were treated with 32.5 µg/mL of both types of NPs for 2, 6 and 24 h. After the incubation cells were centrifuged at 300× *g* for 5 min and supernatants were collected, the concentrations of TNF-α, IL-6, IL-1β, CXCL8, CCL2 and CCL4 in the supernatants were evaluated with a Luminex MAGPIX Multiplex Reader (Merck, Darmstadt, Germany) according to the manufacturer’s instructions, and 100 ng/mL Lipopolysaccharide (LPS) was used as positive control at each time point.

### 2.5. Cellular Uptake of Nanoparticles

#### 2.5.1. CXCL12 NPs Internalization in THP-1 Cells Analysis

THP-1 cells (2 × 10^5^ cells/mL) were collected, resuspended in RPMI-1640 without phenol red (Thermo Fisher Scientific, Waltham, MA, USA) supplemented with 0.5% BSA (Miltenyi Biotec, Bergisch Gladbach, Germany) and incubated 30 min at 37 °C for starvation. After starvation cells were incubated for 45 min at 37 °C with 32.5 µg/mL NPs, then washed at 4 °C. The same experiment was performed with resuspending cells in 10% FBS/medium or 5% HS/medium for 45 min, 2 h and 6 h. The resulting cell fluorescence was analyzed by flow cytometry with MACSQuant Analyzer (Miltenyi Biotec, Bergisch Gladbach, Germany) gating the living cells based on light forward scattering (FSC) and side scattering (SSC). In total, 2 × 10^4^ events per sample were acquired.

#### 2.5.2. CXCR4 Expression

THP-1 cells (2 × 10^5^ cells/mL) were centrifuged at 300× *g* for 5 min, washed twice, resuspended in serum-free medium supplemented with 0.5% BSA (Miltenyi Biotec, Bergish, Germany) and incubated for 30 min at 37 °C for starvation. Then cells were incubated with fluorescently labeled antibodies (APC anti-human CXCR4, Miltenyi Biotec, Bergish, Germany; APC REA control (S) antibodies, Mylteni Biotec, Bergish, Germany) at the manufacturer’s recommended concentration for 30 min on ice in the dark, then washed twice and resuspended in RPMI 1640 without phenol red (Thermo Fisher Scientific, Waltham, MA, USA) at 4 °C. Cell-associated fluorescence was analyzed by flow cytometry with MACSQuant Analyzer (Mylteni Biotec, Bergish, Germany) gating living cells based on FSC and SSC. In total, 3 × 10^4^ events per sample were acquired.

#### 2.5.3. Confocal Microscopy

Living THP-1 cells (2 × 10^5^ cells/mL) were collected and seeded on a glass bottom dish, size 35 × 10 mm glass Ø12 mm (WillCo Wells, Amsterdam, SE, The Netherlands), and incubated for 1 h at 37 °C for adhesion, then washed and incubated at 37 °C for 45 min with 32.5 µg/mL NPs. Cells were washed and incubated in the dark at 37 °C for 15 min with Trihydrochloride Trihydrate (DAPI) (Thermo Fisher Scientific, Waltham, MA, USA) for cell nuclei staining and LysoTracker Green (Thermo Fisher Scientific, Waltham, MA, USA) for lysosomes staining. After incubation, cells were washed and Live Cell Imaging solution (Thermo Fisher Scientific, Waltham, MA, USA) was added. Confocal microscopy images were acquired by a confocal microscope (Leica TCS-SP5) with an oil-immersion 63× objective, 633 nm excitation laser wavelengths and a resolution of 1024 × 1024. Z-sectioning images were acquired with a *z*-slice thickness of about 0.21 µm.

### 2.6. Cell Migration Assay

Chemotaxis was performed in a 24-well plate (Corning, Corning, NY, USA) for 3 h at 37 °C. All samples were resuspended in chemotaxis buffer (RPMI 1640) without phenol red + 0.1% BSA. In total, 200 µL measures of 1 × 10^6^ cells/mL THP-1 were placed onto cell culture inserts with 3 µm pore polyethylene terephthalate (PET) (Meck Millipore, Darmstadt, Germany). Bottom wells were loaded with 800 µL of the different NP suspensions at the final NP concentrations described in the text, 10 nM human CXCL12 (Chemotactics, San Diego, CA, USA) or chemotaxis buffer, depending on the experiment. Where indicated, THP-1 was pretreated with CXCL12-NPs or non-functionalized NPs for 45 min. After 3 h incubation at 37 °C, filters were removed and 400 µL suspensions from the bottom wells were read by flow cytometry. Samples were counted through BD FACSDiva software 6.0 provided by BD Biosciences (San Jose, CA, USA), gating the living cells based on FSC and SSC. In total, 1.5 × 10^4^ events per sample were acquired.

### 2.7. Statistical Analysis

Data were expressed as mean ± standard deviation (SD). For statistical analysis, GraphPad Prism 8 software was used (San Diego, California, CA, USA). *p*-values were calculated using unpaired *t* test with Welch’s correction **** *p* < 0.0001.

## 3. Results

### 3.1. Nanoparticle Sysnthesis and Physicochemical Characterization

#### 3.1.1. Formulation and Characterization of Biotinylated PLGA/Pluronic Nanoparticles

PLGA NPs were prepared via microfluidics-assisted nanoprecipitation by mixing a solution of the polymer in acetone with that of a surfactant (Pluronic F127) in water. The procedure has been previously optimized [43] and allows the preparation of Pluronic-coated NPs to proceed in a very reproducible and operator-independent fashion. When Pluronic bears functional termini, the NPs are decorated with known densities of, e.g., targeting peptides [42,44]. In this work, we have employed biotinylated Pluronic F127 (F127-BIO) to provide a flexible strategy of NP decoration: the Pluronic biotin groups are located at the termini of the hydrophilic blocks, thus they end up decorating the NP’s surface when Pluronic molecules adsorb onto the PLGA to stabilize its interface with water. Separately, we have complexed a mixture of biotin, a biotinylated fluorophore (atto 610) and biotinylated human CXCL12 with streptavidin, using stoichiometric ratios between streptavidin and total biotins, which led to the occupation of three out of the four streptavidin interaction sites. The resulting complexes therefore still have one streptavidin site available for complexation, which is then used to bind the biotin groups on the NP surfaces; if the streptavidin would be presented to particles with more than one free site, the latter may agglomerate, or streptavidin may end up with multiple anchoring points, leading to a different peptide presentation. In short, each biotin on the particle surface links to only a single streptavidin-based functional cluster; each cluster contains one fluorophore and at least one biotin, while CXCL12 is present only in about 10% of the clusters (Figure 1B).

The biotinylated Pluronic was prepared by reacting Pluronic with divinyl sulfone (converting 63% of OH into vinyl sulfones), and then submitting the product first to the Michael-type addition of cysteamine and then to the DMTMM-mediated amidation with biotin (Figure 1A top). The ^1^H NMR showed the complete disappearance of the olefin resonance of the vinyl sulfone and the presence of biotin, with an overall conversion of 55% of the Pluronic hydroxy groups (Figure 1A bottom). F127-BIO was present on the NP’s surface alone or in a mixture with unmodified F127, which allows one to tune the surface density of functional groups. The morphology of the NPs was evaluated by cryoEM, confirming the spherical shape and homogeneity of the particle size (Figure 1C). When analyzed via DLS in batch mode, the formulations had a Z-average hydrodynamic size between 85 and 90 nm and narrow polydispersity (Figure S1). Asymmetric flow field-flow fractionation (AF4) analysis with DLS, static light scattering (SLS) and concentration detectors allows a more precise evaluation of the nanoparticle dimensions, measuring the radius of gyration (Rg) and the hydrodynamic radius (Rh) separately on samples fractionated on the basis of their dimensions (Figure S2); through AF4, the average hydrodynamic size turns out to be about 10–20 nm smaller than the one measured in the batch where the scattering is predominantly influenced by larger particles. Finally, a shape factor (defined as the ratio between the radius of gyration and the hydrodynamic one) around 0.5–0.6 was observed, indicating a core-shell structure of the particles where a dense core of PLGA is coated with a hydrated layer of Pluronic. In terms of stability, despite being based on a biodegradable polymer, no difference in size was observed upon storage for up to two months at 4 °C, thereby ensuring a long shelf life of the formulations (Table S1).

#### 3.1.2. Streptavidin-Mediated Decoration of Biotinylated PLGA/Pluronic Nanoparticles

We confirmed by 2-anilinonaphthalene-6-sulfonic Acid (ANS) assay the accessibility of the biotin bound on PLGA NPs to streptavidin (Figure S3, left). Then, different formulations were screened by titrating the NPs coated with a blend of F127-BIO and F127 (with 25, 50 and 100% weight ratio) with streptavidin (Figure S3, right). Please note that in these experiments, the streptavidin was pre-saturated with three equivalents of biotin (no fluorophore, no peptide, but still a single active site on the protein). The formulation with a 25:75 F127-BIO/F127 weight ratio and a 1:2 streptavidin/biotins on F127-BIO was selected, which corresponds to 10 nmol of streptavidin per mg of NPs. The sample was further analyzed by AF4 coupled with SLS and DLS. No change in Rh of the control and streptavidin-conjugated sample was observed (Figure 1D). Moreover, AF4 can in principle detect the presence of free streptavidin, but its amount was under the detection limit, ensuring the virtually quantitative yield of the coupling (inset in Figure 1D).

### 3.2. Detection of CXCL12 on the Nanoparticle and CXCL12-NP Characterization

**Figure 2:**
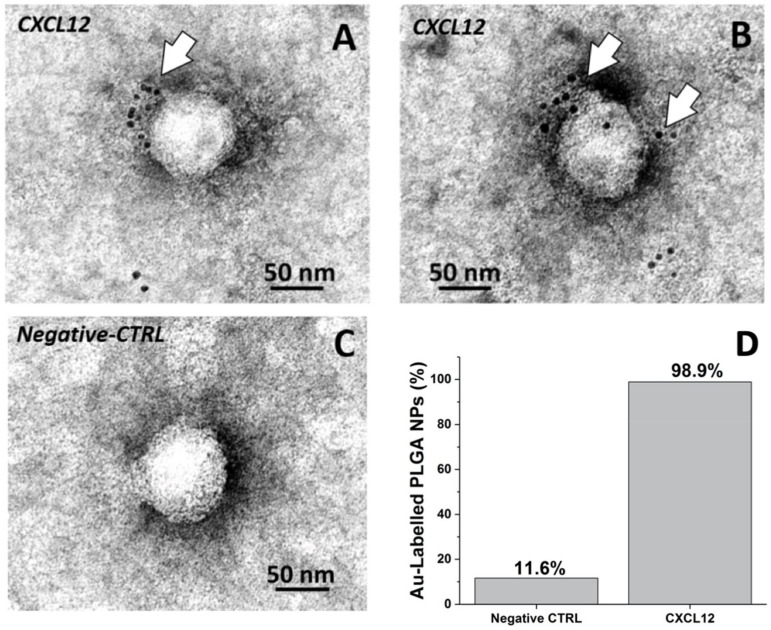
Negative staining EM immunolabeling. Representative images of treated (CXCL12) (**A**,**B**) and negative control (Negative CTRL) CXCL12-NPs (**C**). White arrows point to the Au nanoparticles conjugated to the secondary antibody decorating the NP surface. (**D**) Columns show the percentage of Ig-Au-decorated CXCL12-NPs in treated and negative control samples.

After NP decoration we confirmed CXCL12’s presence on the NP surface (Figure 2). Epitope mapping of CXCL12 by negative staining EM immunolabeling with specific anti-CXCL12 primary antibody and Au-conjugated secondary antibody (see Matherial and Methods) was performed. Figure 2 shows the negative staining projection images of immunogold-labeled CXCL12-decorated PLGA NPs. As expected, IgG-Au localizes on the CXCL12-NP surface (compare Figure 2A,B with Figure 2C). This result is highlighted by the quantification of the Au decorated nanoparticles (Au-PLGA) number in treated and control samples, as reported in Figure 2D.

In total, 32.5 µg/mL measures of CXCL12-NPs were further characterized by analyzing the Z-average size and Z-potential by DLS. This concentration of CXCL12-NPs corresponds to 10 nM of NP surface-bound chemokine in the final suspension. The chemokine presence on the NP surface did not affect the NP size, but induced a charge inversion which we ascribe to the presence of CXCL12 on the NP surface (isoelectric point of CXCL12 ≈ 9 (Table 1) [45,46]. This result further demonstrated the efficient CXCL12 functionalization of PLGA NPs.

### 3.3. Biocompatibility of CXCL12-NPs

#### 3.3.1. Toxicity

**Figure 3:**
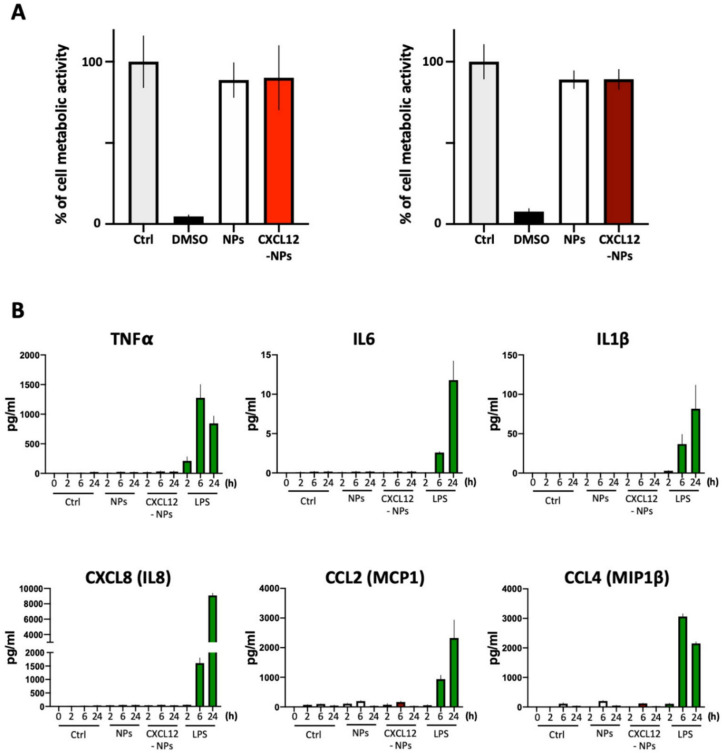
(**A**) Histograms of metabolic activity of THP-1 in two sera supplemented media. Untreated THP-1 cells (Ctrl, light gray bar), THP-1 cells treated with non-functionalized NPs (white bar), CXCL12-NPs (light red bar) in 10% FBS/medium (left graph), CXCL12-NPs (dark red bar) 5% HS/medium (right graph). Ten percent DMSO (black) was used as positive control. Data are represented in percentage relative to untreated control. (**B**) Cytokine release. Column graphs show the concentrations of TNF-α, IL-6, IL-1β, CXCL8, CCL2 and CCL4 released by THP-1 cells in 5% HS/medium, after treatment with non-functionalized NPs (NPs), CXCL12-NPs or 100 ng/mL LPS for 2, 6 and 24 h, measured by Bio-Plex MAGPIX Multiplex Reader. Control (Ctrl) represents untreated cells. Results are expressed in pg/mL. All bars represent at least three independent experiments +/− SD.

To evaluate the possible cytotoxic effects of CXCL12-NPs we performed a WST-8 assay (Figure 3A). The THP-1 cells were cultured in the presence of 32.5 µg/mL NPs for 24 h in two different media. CXCL12-NPs, as well as non-functionalized control NPs, did not induce a significant reduction of the metabolic activity of cells in culture medium supplemented with 10% FBS (Figure 3A, left graph), nor in 5% HS (Figure 3A, right graph). The 10% DMSO was used as positive control for cell death, whereas THP-1 in the absence of NPs was considered the negative control of the test (100% viability). We did not measure longer time points since after 24 h THP-1 replication occurs, and this proliferation would reduce the concentration of NPs per single cell.

#### 3.3.2. Inflammatory Cytokine Release

To develop NPs for biomedical applications, toxicity assays may not be sufficient to predict potential nocious effects. Cell exposures to NPs could induce inflammatory signals, eliciting an immune response. One of the triggering events of inflammation is mediated by the release of specific cytokines. We treated THP-1 with 32.5 µg/mL CXCL12-NPs or non-functionalized NPs for 2, 6 and 24 h following the release of six different inflammatory mediators, namely TNFα, IL6, IL1β, CXCL8, CCL2 and CCL4 (Figure 3B). The first three cytokines activate several pathways leading to innate immune response, whereas CXCL8, CCL2 and CCL4 mainly induce phagocyte (i.e., neutrophils, monocytes/macrophages) recruitment. Cytokine detection was performed in a multiplex system and THP-1s were cultured only in HS/medium. The experimental restriction to this medium was due to the presence of possible costimulatory molecules in humanized medium, which are not present in the sera from other species. No significant release of inflammatory cytokine was detected at any time point (Figure 3B and Figure S4). Although unstimulated THP-1s in 5% HS culture medium express chemokines at low levels, CXCL12-NPs did not modify their concentration in the medium. THP-1s were stimulated with 100 ng/mL LPS to observe the substantial presence of cytokines in the medium. These results demonstrate that CXCL12NPs are not cytotoxic and do not induce inflammatory cytokine release in vitro.

### 3.4. CXCL12-NP Internalization in THP-1 Cells

**Figure 4:**
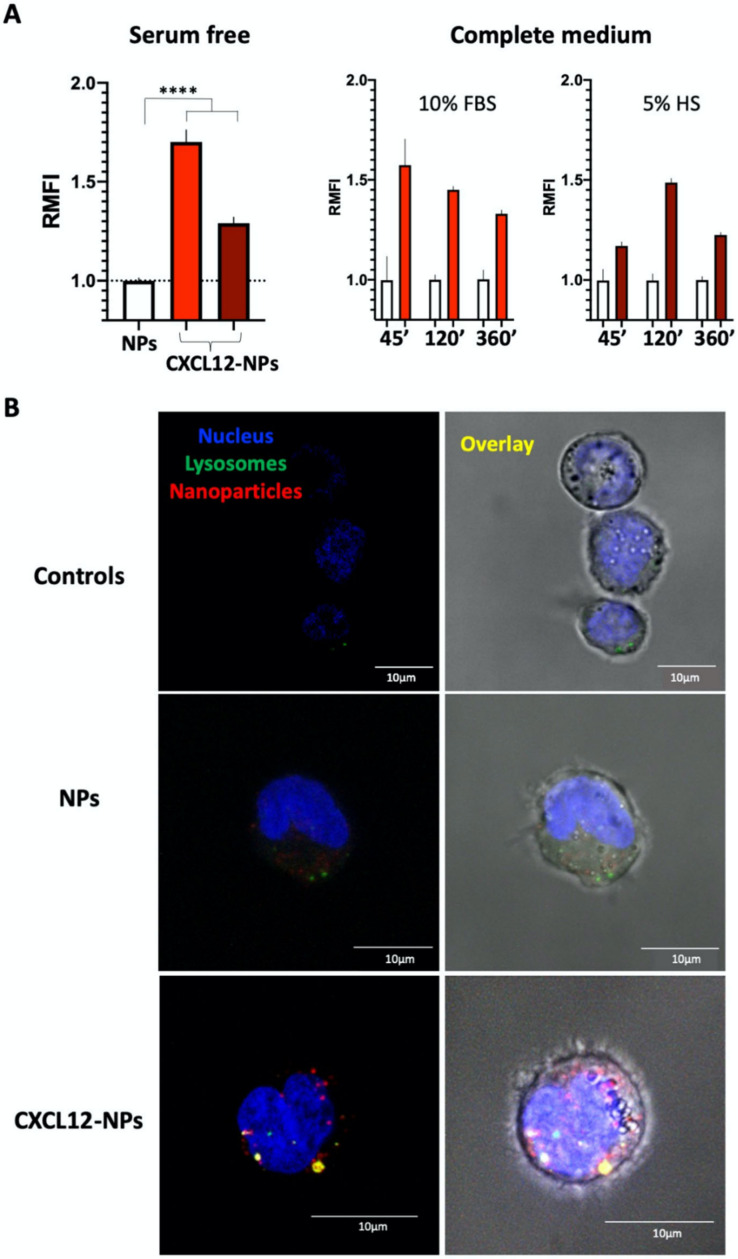
(**A**) CXCL12-NPs internalization in THP-1 cells cultured in 10% FBS/medium (red column) or 5% HS/medium (dark red column) after 45 min of NP administration in serum-free conditions (left graph) and after 45 min, 2 h and 6 h in both media conditions (10% FBS, center graph; 5% HS, right graph). Bars represent the relative median fluorescence intensity (RMFI) of at least three independent experiments +/− SD. Statistically significant differences were determined by unpaired *t* test with Welch’s correction **** *p* < 0.0001. (**B**) Confocal images of 10% FBS/medium-cultured THP-1 cells. Untreated cells (controls), cells treated for 45 min with non-functionalized NPs (NPs) and CXCL12-NPs. DAPI (blue) represents cell nucleus, Lyso-tracker (green) represents lysosomes and in red we show the Atto610 fluorescence of labeled NPs and CXCL12-NPs. The overlapping (yellow) of Atto610 (red) and LysoTracker (green) indicates nanoparticle co-localization with the lysosomes. Scale bar = 10 μm.

CXCL12-NP binding and internalization into THP-1 cells was analyzed by flow cytometry and confocal microscopy (Figure 4).

Firstly, we measured THP-1 uptake in the presence of NPs after 45 min in serum-free medium. Cells previously cultured in FBS conditioned medium showed a >1.7 times increased internalization vs. the non-functionalized NPs (Figure 4, left graph). The uptake of CXCL12-NPs in the THP-1 grown in HS/medium was also higher than control NPs, though the increment was less pronounced (roughly 1.3 times). It is worth mentioning that the CXCR4 expression of THP-1 in 10% FBS/medium was higher than in 5% HS/medium, as shown by flow cytometry in Figure S5. We cannot assert that higher CXCR4 expression is the only event increasing CXCL12-NP internalization, but it could contribute to the highest NP chemokine uptake at 2 h in the FBS medium. As already show in our previous work for a different chemokine-decorated particle [41], we confirmed CXCL12-NP/CXCR4 binding (Figure S6) by inhibition of CXCL12-NP internalization. The THP-1 cells were pretreated with free CXCL12 for 45 min before performing NP internalization. We observed that 1 nM CXCL12 was sufficient to drastically reduce the uptake of CXCL12-NP in CXCR4^hi^ THP-1 in FBS/medium. Interestingly, the facilitated uptake of CXCL12-NPs vs. “naked” NPs was not constant over time (Figure 4A, center and right graphs). While in the FBS/medium-cultured cells, CXCL12-NP internalization reached the highest increase in 45 min and slowly decreased with time (Figure 4A center graph), in HS/medium, CXCL12-NPs showed a major difference from non-functionalized NPs at 2 h (Figure 4A, right graph).

As proven by confocal microscopy (Figure 4B), most of the CXCL12-NPs were visible inside the cell.

In FBS/medium, some particles colocalized with lysosomes after only 45 min (Figure 4B). On the other hand, in HS-supplemented medium, most of the CXCL12-NPs were found close to the nucleus with minor colocalization with lysosomes (Figure S7).

### 3.5. CXCL12-NPs Do Not Induce THP-1 Migration

**Figure 5:**
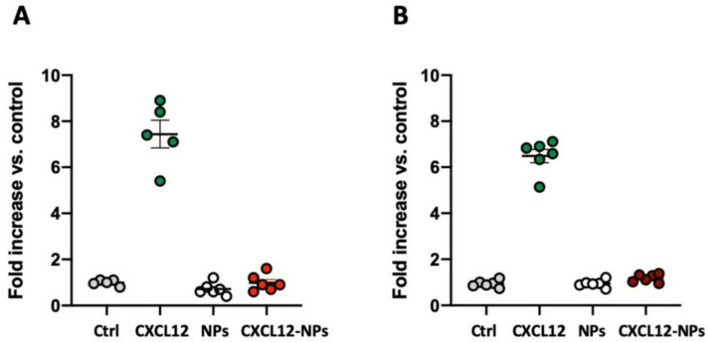
THP-1 cells’ migration towards non-functionalized NPs and CXCL12-NPs in both media conditions: (**A**) 10% FBS/medium; (**B**) 5% HS/medium. In total, 10 nM of CXCL12 was used as positive control. Scatter dot plots represent the fold increase of at least five independent experiments +/− SEM, each dot representing the average of a triplicate.

CXCR4 internalization requires chemokine-induced signal transduction, however this pathway may not be related to the activation of downstream kinases or cytoskeleton reorganization when different ligands bind the same receptor [26,47,48,49,50]. We tested our NPs as chemoattractants for THP-1 to assess whether the marked CXCL12-NP internalization would lead to chemotaxis. As shown in Figure 5, CXCL12-NPs at 32.5 µg/mL (equivalent to 10 nM bound CXCL12) were not able to induce THP-1 migration in either FBS/medium- or HS/medium-cultured cells (Figure 5A,B). Curiously, free CXCL12 (positive migration control) induced THP-1 chemotaxis with similar efficiency in both media, although the THP-1 expression of CXCR4 was higher in FBS/medium (Figure S5). A dose–response analysis was also performed and we observed the potential U-shaped curve of CXCL12-NPs inducing cell migration at lower NP concentrations (Figure S8). No statistically significant migration was induced by CXCL12-NPs at 16.1 µg/mL, nor at 3.3 µg/mL.

This result proves that CXCL12-NPs have an agonistic activity on CXCR4, but their internalization does not lead to CXCR4-mediated chemotaxis, possibly eliciting a biased CXCL12/CXCR4 signaling pathway.

### 3.6. CXCL12-NPs Prevent CXCR4 Mediated Cell Migration

**Figure 6:**
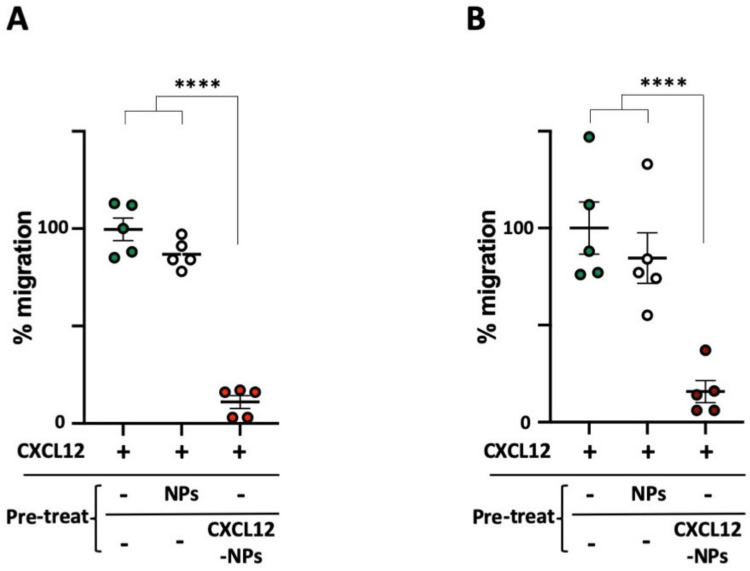
Cell migration towards 10 nM human CXCL12, after the treatment with 32.5 µg/mL non-functionalized NPs (white dots) and CXCL12-NPs (red dots) in both (**A**) 10% FBS/medium and (**B**) 5% HS/medium. Data are shown in percentage relative to untreated THP-1 cell migration and represent the average of at least five independent experiments +/− SEM. All the experiments were performed in triplicate. Statistically significant differences were determined by unpaired *t* test with Welch’s correction **** *p* < 0.0001.

The results reported so far demonstrate that CXCL12 on the PLGA/Pluronic NP surface facilitates the uptake of the particles in CXCR4^+^ cells without inducing THP-1 chemokine-mediated migration. We then investigated if, otherwise, CXCL12-NPs could antagonize CXCL12-induced chemotaxis. The 45 min pre-administration of CXCL12-NPs abrogated THP-1 migration towards CXCL12, lasting for 3 h, as shown in Figure 6. The chemotaxis of cells cultured in FBS/medium (Figure 6A) or in HS/medium (Figure 6B) was dramatically reduced by a similar extent. Remarkably, the treatment with non-functionalized NPs did not impair cell migration, emphasizing the role of the chemokine on the NP’s surface. The specificity of CXCL12-NPs for CXCR4-mediated chemotaxis was further confirmed via chemotaxis experiments of THP-1 migrating against a different chemokine, namely CCL5 (Figure S9). CXCL12-NPs do not inhibit the CCL5/CCR5-induced chemotaxis of THP-1, proving their specificity for CXCR4-mediated signaling. The blockade of CXCL12-induced chemotaxis demonstrates the potential role of CXCL12-NPs as antagonists for CXCR4^+^ migrating cells.

## 4. Discussion

CXCL12/CXCR4 signaling represents an important delivery target for cancer cells [5,51]. Its role in the migration and localization of metastatic cells has been known for almost twenty years [52,53], and it is gaining more and more importance in calibrating therapies with CXCR4 antagonists and chemotherapeutic drugs. To achieve significant benefits, these treatments need the long-term or chronic administration of the therapy, and adverse effects can occur.

We believe that CXCL12-PLGA/Pluronic NPs may show interesting features in developing novel biodegradable antagonists and drug carriers. Our particles were highly internalized in CXCR4^+^ cells, and did not induce chemotaxis, but, on the contrary, blocked CXCR4^+^ cell migration. Furthermore, in the presented experimental conditions, CXCL12-NPs were not toxic or immunogenic, and their biodegradable polymeric composition could allow drug loading. In addition, PLGA/Pluronic NPs manufactured through microfluidics-assisted nanoprecipitation with a consolidated protocol [43] display highly reproducible features, such as, size, shape and stability. The precise structure and polymer composition permit wide flexibility in chemical modification, such as was recently proven for decorating the particles with arginine-glycine-aspartic acid (RGD) peptides to exploit integrin-mediated cancer cell targeting [42].

The possibility of selectively driving polymeric NPs in CXCR4^+^ cells and stopping their migration without other agonistic activity offers some advantages with respect to small-molecule CXCR4 antagonists, such as AMD3100 [54] or MSX-122 [55], which show partial inhibition of CXCL12/CXCR4 signaling. As well, short peptides derived from truncations of the chemokine N-terminal part cannot assure the receptor-binding stability of the entire CXCL12. The functionalization of NP surfaces with the mentioned chemical moieties has been already applied in different drug delivery systems for CXCR4^+^ cancer cells [5]. Unfortunately, the wide expression of CXCR4 and its several roles played in many tissues increase the possibility of mistargeting, and the consequent unexpected side effects. Although our results are limited to in vitro models, we demonstrated that our biocompatible CXCL12-PLGA/Pluronic NPs specifically block CXCR4^+^ cell migration. Actually, as shown by the specific inhibition of CXCR4-induced chemotaxis without affecting the different chemokine-induced signaling (i.e., CCL5/CCR5), and the co-localization of CXCL12-NPs with lysosomes, we are able to speculate on the possible sequestration of CXCR4 bound to the CXCL12-particle, and its impaired or limited release from it. However, this condition might be temporary considering the biodegradability of the NP polymers. Advanced cell models will be required in further experiments to prove these hypotheses.

It will also be interesting to understand if other intracellular signaling pathways, independent of chemotaxis, could be triggered by our particles. For example, CXCL12-NPs show CXCR4 internalization, but no information on possible kinase activity or intracellular calcium mobilization is available yet. Another crucial aspect to be investigated is the possible targeting interference due to protein corona. However, our experiments have been already performed in two different sera-conditioned media, including human serum-supplemented THP-1 cell cultures. The obtained results did not show significant differences in the chemotaxis, even if the CXCL12-NP internalization was more pronounced in FBS/medium, as well as the CXCR4 expression on the THP-1 cells cultured in this condition.

Our future aim will be the use of our particles to stop metastatic cells in cancer models reducing the adverse effects of the common chemotherapeutic drugs. Of course, further focused research will be needed to see CXCL12-NPs’ potential in vivo.

## 5. Conclusions

We decorated with CXCL12 the surface of PLGA/Pluronic NPs synthesized by the microfluidics-assisted protocol. As regards non-functionalized NPs, CXCL12-NPs showed increased internalization into CXCR4^+^ THP-1 cells cultured in different media conditions without inducing their chemotaxis. On the contrary, our particles were able to stop CXCR4-mediated migration. The biodegradable polymer composition and the demonstrated biocompatibility suggest CXCL12-PLGA/Pluronic NPs as suitable candidates to develop novel antagonists for metastatic cell migration.

## Figures and Tables

**Table 1:** Z-average size, polydispersity index (PDI) and Z-potential of non-functionalized NPs and CXCL12-NPs. PDI < 0.2 means that nanoparticles are monodisperse and measures are accurate.

	Z-Average Size (nm)	PDI	Zeta Potential (mV)
Non-functionalized NPs	90 ± 5	0.12 ± 0.04	−10 ± 3
CXCL12-NPs	90 ± 5	0.10 ± 0.03	20 ± 2

## Supplementary Materials

The following are available online at https://www.mdpi.com/2079-4991/10/11/2304/s1, Figure S1: Nanoparticle size and stability, Figure S2: Nanoparticle AF4 analysis, Figure S3: Nanoparticle biotin/streptavidin mediated decoration, Figure S4: Cytokine release induced by NPs and CXCL12-NPs, Figure S5: CXCR4 expression on THP1 cell membrane in different serum conditioned media, Figure S6: Free CXCL12 pretreatment inhibits CXCL12-NPs internalization, Figure S7: CXCL12-NPs internalization in THP1 cells cultured in 5% Human Serum supplemented medium, Figure S8: Chemotaxis with lower NP concentrations, Figure S9: CXCL12-NPs specifically inhibit CXCR4-mediated THP1 migration and not against different chemokines/chemokine receptors like CCL5/CCR5.
