# Supplementary material for: CXCL12-PLGA/Pluronic Nanoparticle Internalization Abrogates CXCR4-Mediated Cell Migration
Source: Nanomaterials (Basel). 2020 Nov 20;10(11):2304. doi: 10.3390/nano10112304 (PMC7699919; doi:10.3390/nano10112304)
Supplement: Supplementary file 1 [file nanomaterials-10-02304-s001.pdf]

# *Supplementary Information*

## Nanoparticle size and stability

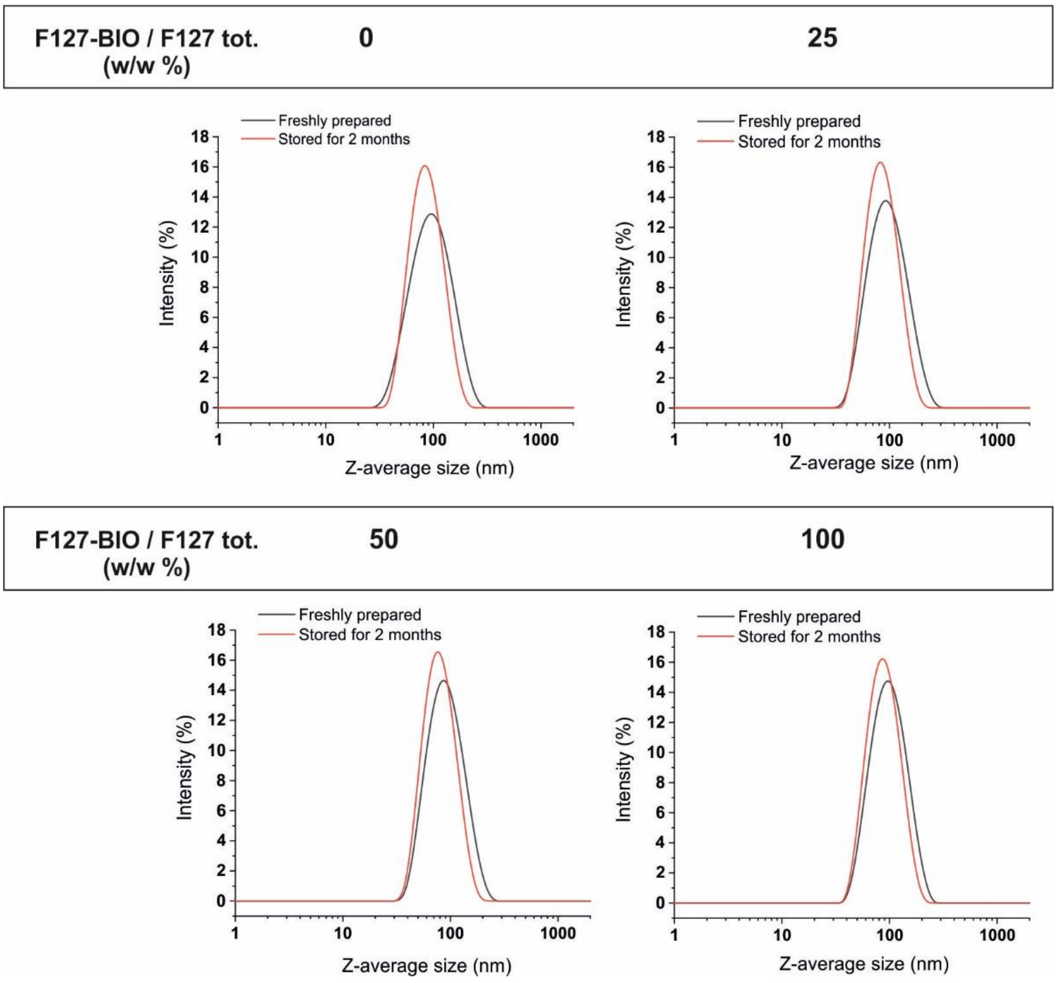


**Figure S1**. Size distribution obtained by DLS of a 0.5 mg/mL suspension of PLGA NPs in water after preparation and after 2 months of storage at 4 °C. All nanoparticles were prepared in a microfluidic reactor and coated with a blend of Pluronic F127 and Pluronic F127-BIO at different weight ratio.

**SI Table 1.** DLS analysis of PLGA NPs in water after preparation and after storage.

| **F127-BIO / F127 tot. (w/w %)** | **Freshly prepared** | | **Stored for 2 months** | |
|---|---|---|---|---|
| | **Z-average size (nm)** | **PDI** | **Z-average size (nm)** | **PDI** |
| 0 | 87 | 0.14 | 80 | 0.10 |
| 25 | 88 | 0.13 | 80 | 0.11 |
| 50 | 84 | 0.11 | 75 | 0.13 |
| 100 | 91 | 0.11 | 83 | 0.11 |

## Nanoparticle AF4 analysis

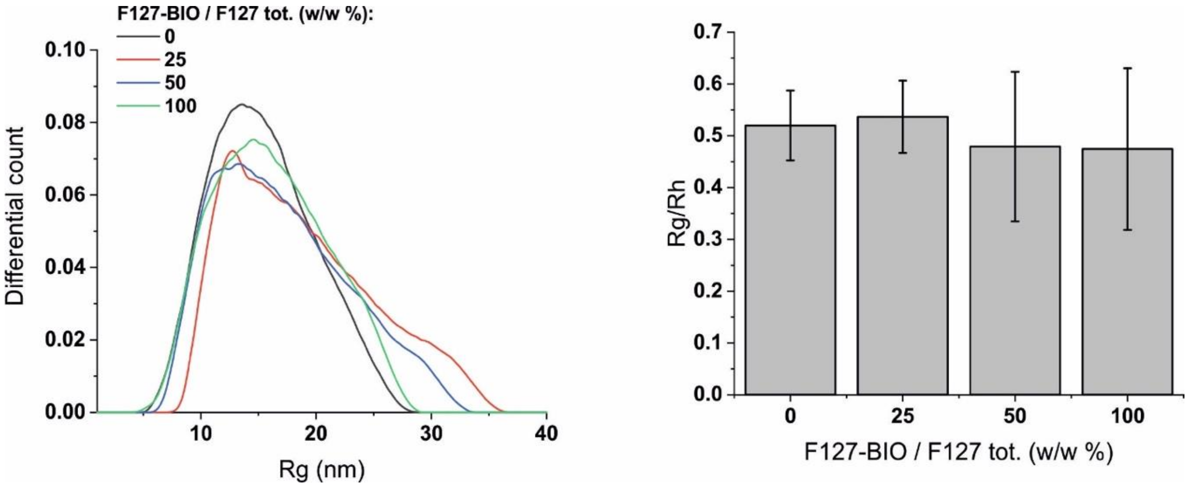


**Figure S2.** Nanoparticle analysis by asymmetric flow field-flow fractionation (AF4). *Left:* Radius of gyration (Rg) differential distribution obtained for PLGA NPs stabilized with a mixture of unmodified and biotinylated Pluronic F127. *Right:* summary of the results obtained for the Rg/Rh ratio (the average and standard deviation of all the Rg/Rh values recorded throw-out the peaks are reported).

## Nanoparticle biotin/streptavidin mediated bioconjugation

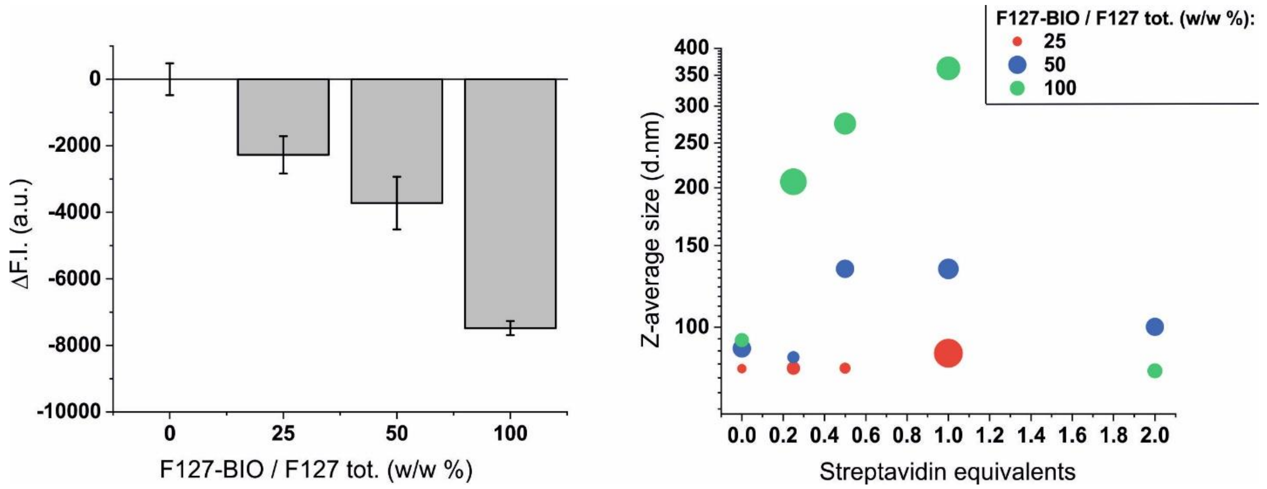


**Figure S3**. Right: Competitive binding assay for Pluronic F127-BIO. PLGA NPs prepared with different ratios between unmodified and biotinylated Pluronic F127 were incubated with a mixture of streptavidin and ANS (2-Anilinonaphthalene-6-Sulfonic Acid). ANS is a fluorophore that has large increase in quantum yield in the presence of streptavidin. The biotin present on the surface of PLGA NPs caused the displacement of the bound fluorophore with concomitant quenching of the fluorescence compared with the control with unmodified surfactant (F127-BIO 0%). Left: Z-average size measured by DLS of biotinylated PLGA NPs mixed with different amounts of streptavidin (previously quenched with 3 equivalents of biotin). The size of the circles refers to the PDI of the distributions that ranged between 0.1 and 0.45.

## Cytokine release induced by NPs and CXCL12-NPs

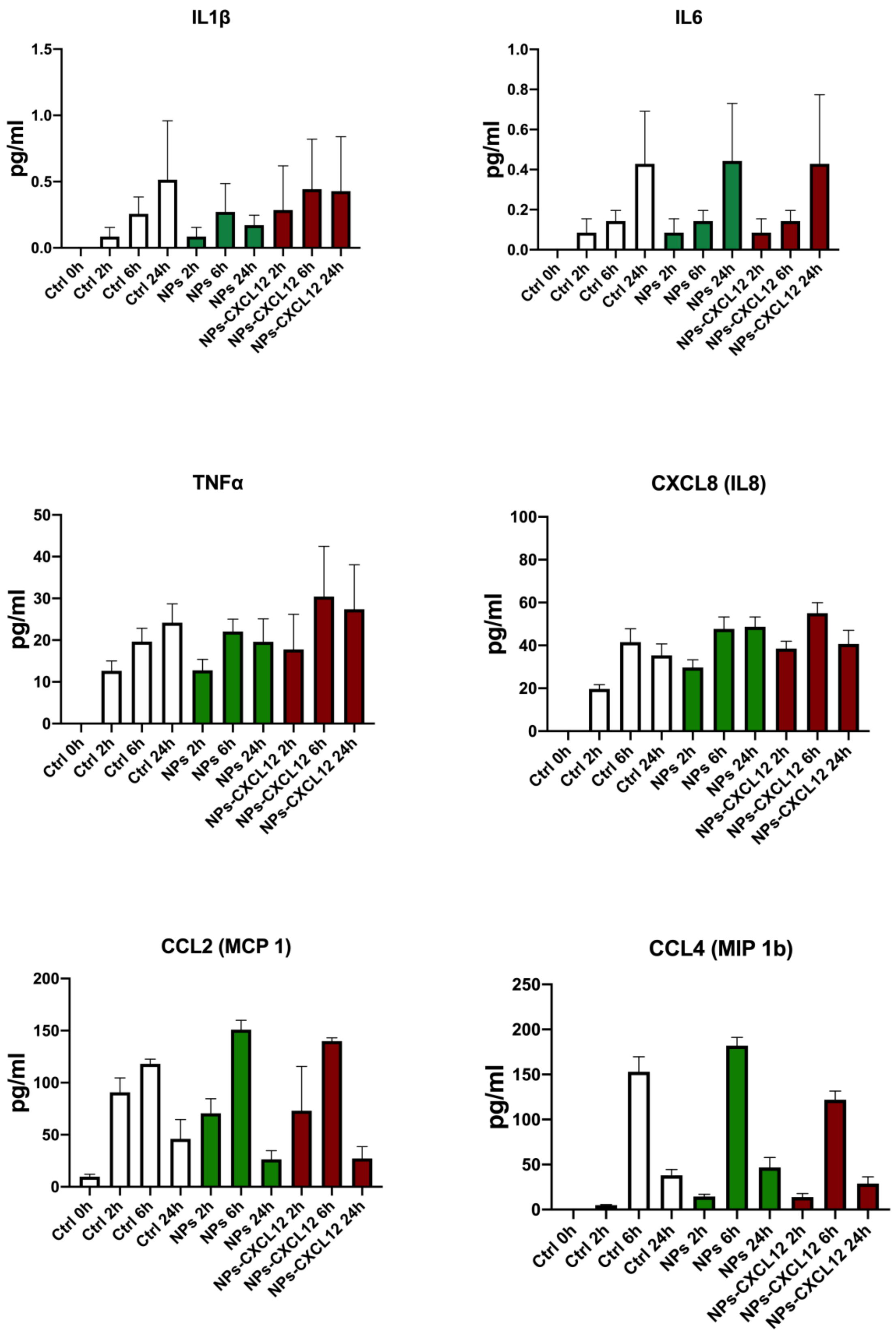


**Figure S4**. Cytokine release in linear scale of THP-1 treated with control NPs and CXCL12-NPs.

## CXCR4 expression on THP1 cell membrane in different serum conditioned media

A

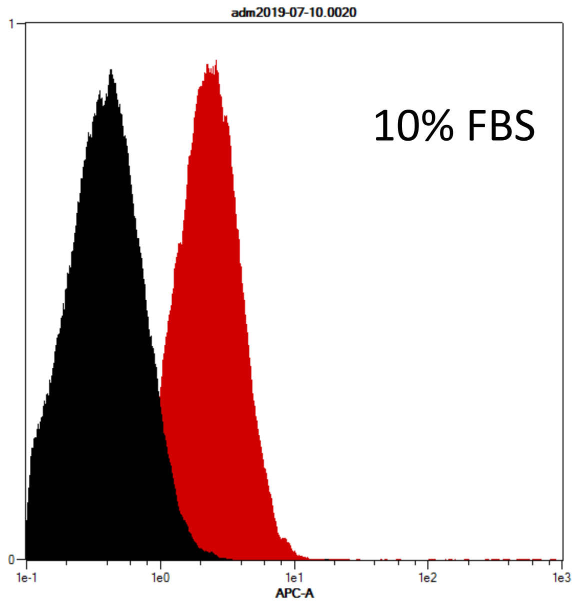


B

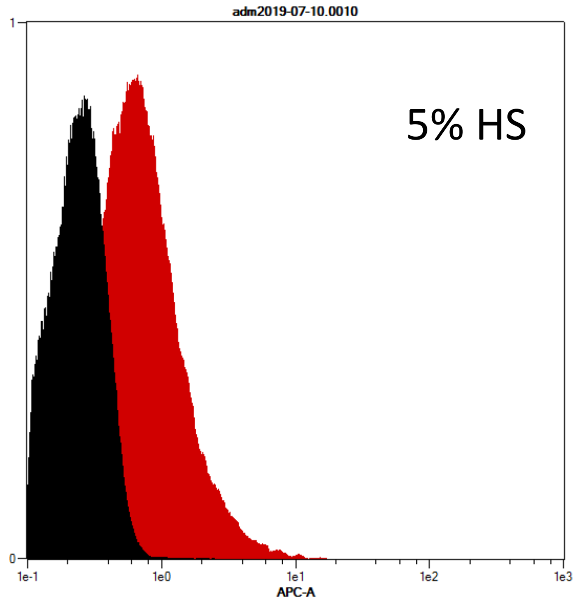


**Figure S5**. CXCR4 expression on THP-1 surface cultured in 10% FBS/medium (SI Figure 4A) and 5% HS/medium (SI Figure 4B). Histograms show antibody fluorescence on linear scale. APC anti-human CXCR4 (red) and APC isotype (black), indicating CXCR4 and isotype control expression, respectively. Receptor staining has been performed after 30 min of THP-1 starvation in 0.5 % BSA supplemented media.

**Free CXCL12 pretreatment inhibits CXCL12-NPs internalization**

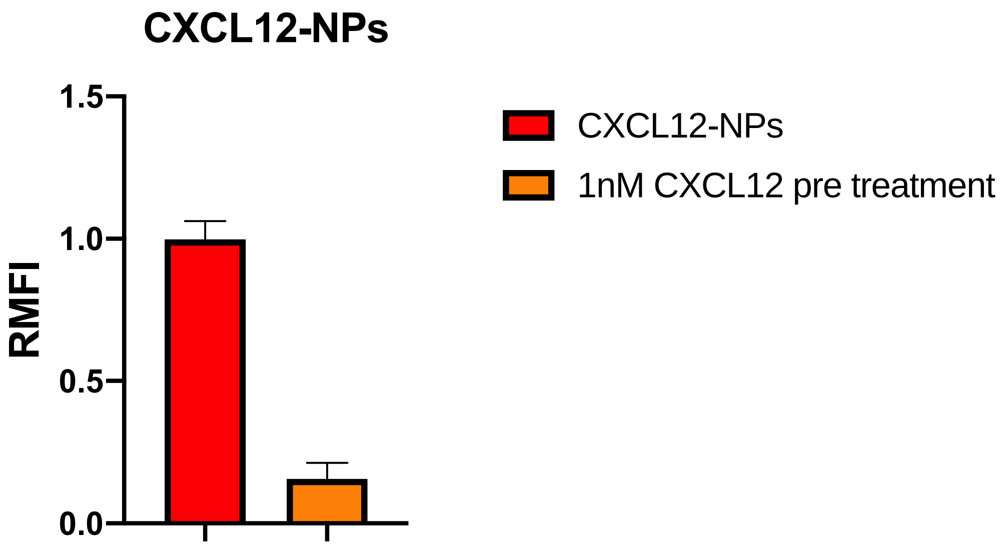


**Figure S6**. Internalization of 32.5 μg/mL CXCL12-NPs in 10% FBS/medium cultured THP-1 cells pretreated or not with 1nM CXCL12. Bars represent the relative median fluorescence intensity (RMFI) of at least three independent experiments +/- SD (error bars).

**CXCL12-NPs internalization in THP1 cells cultured in 5% Human Serum supplemented medium**

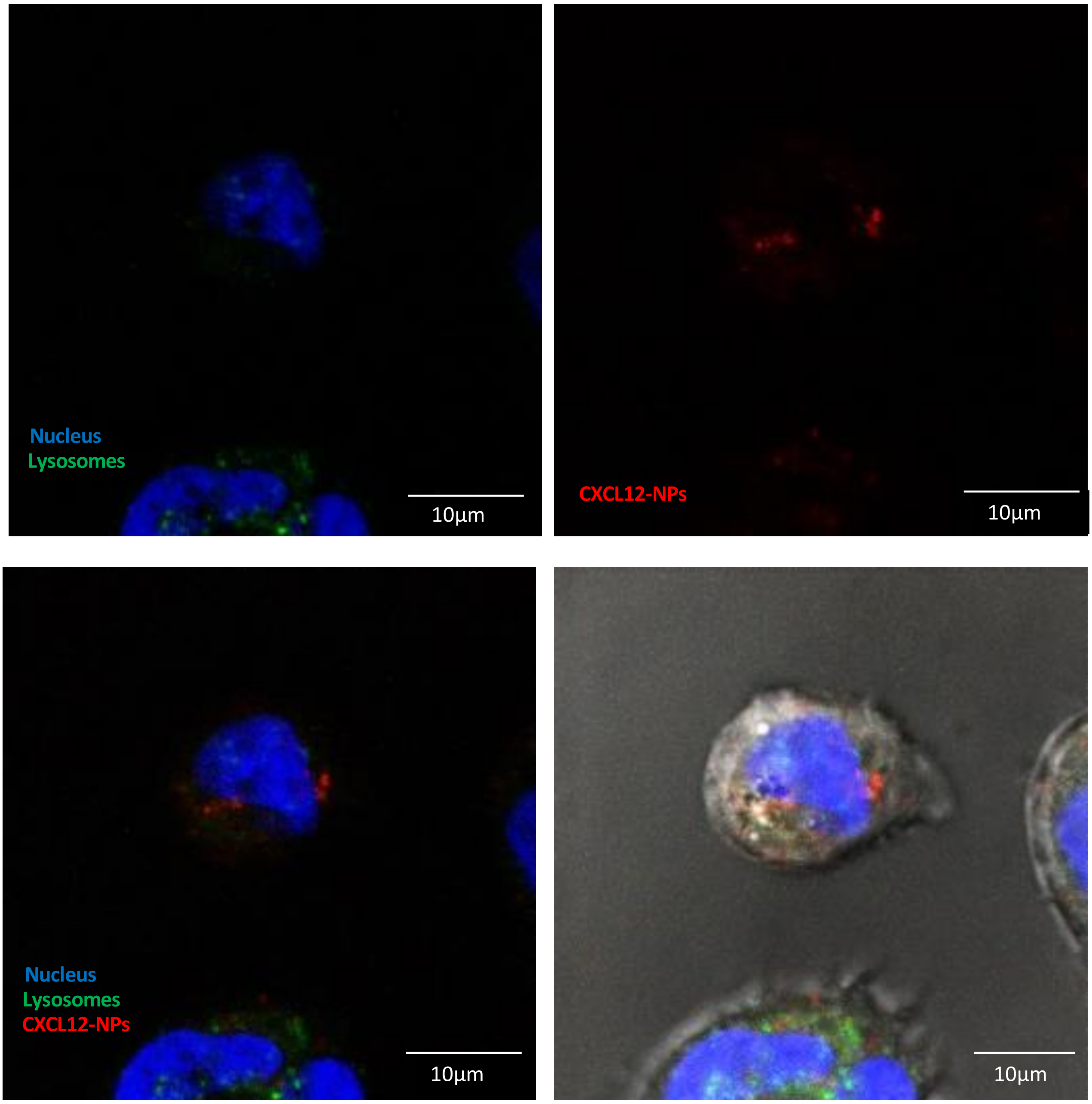


**Figure S7**. Z maximum projection of THP-1 cells cultured 5% HS/medium and treated with CXCL12-NPs. DAPI (blue) represents cell nucleus, Lyso-tracker (green) represents lysosomes and in red the Atto610 fluorescence of labelled CXCL12-NPs.

## Chemotaxis with lower NP concentrations

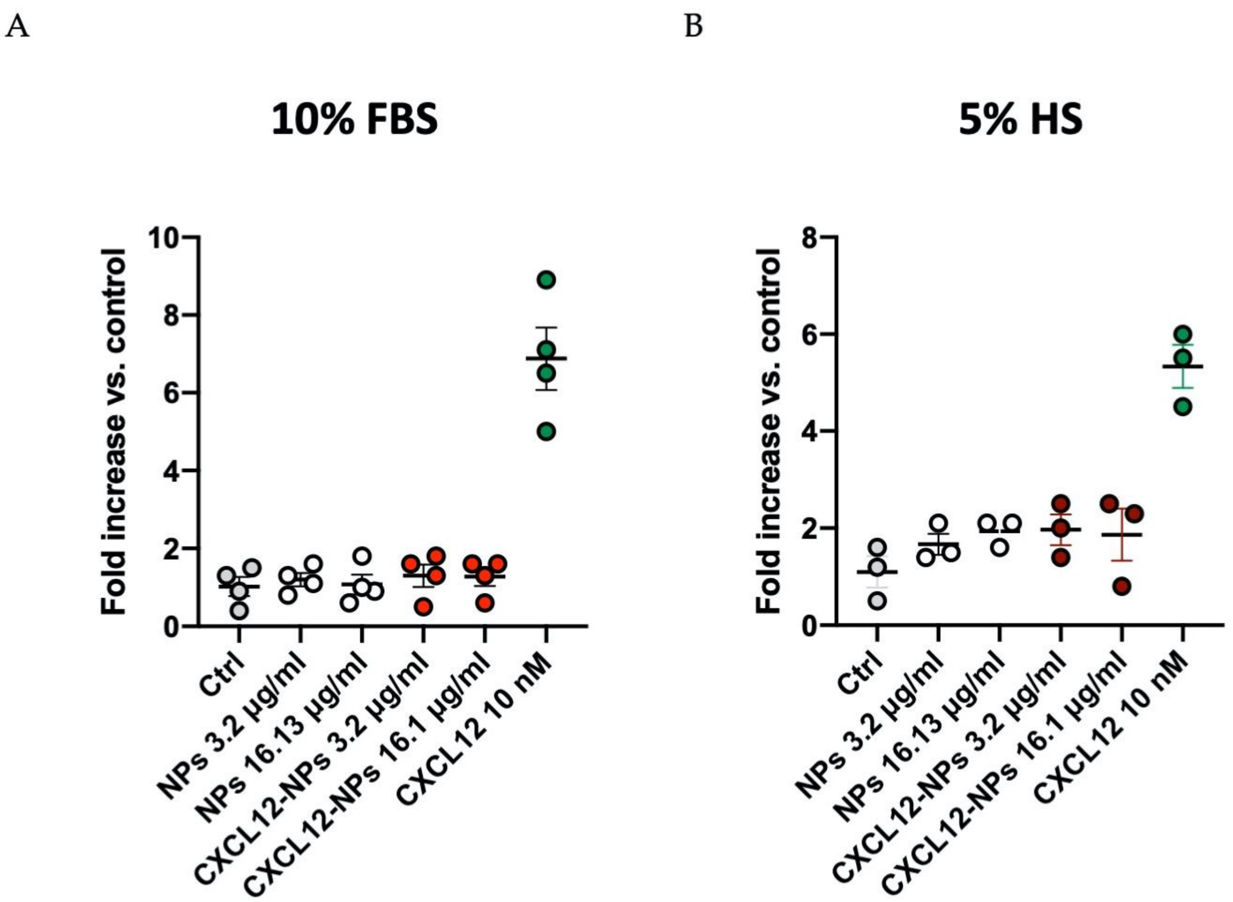


**Figure S8**. THP-1 cells migration towards non-functionalized NPs and CXCL12-NPs in both media conditions A) 10% FBS/medium; B) 5% HS/medium. 10nM CXCL12 was used as positive control. Scatter dot plots represents the fold increase of four (FBS) and three (HS) independent experiments +/- SEM, each dot representing the average of a triplicate.

**CXCL12-NPs specifically inhibit CXCR4-mediated THP1 migration and not against different chemokines/chemokine receptors like CCL5/CCR5**

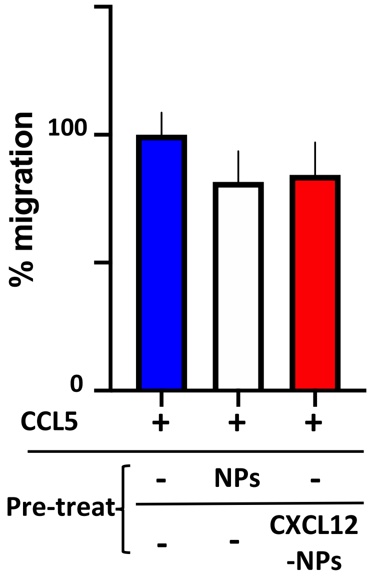


**Figure S9.** Cells migration towards 10 nM human CCL5, after the treatment with 32.5 µg/ml non-functionalized NPs (white column) and CXCL12-NPs (red column) in 10% FBS/medium. Data are shown in percentage relative to untreated THP-1 cell migration and represent the average of at least three independent experiments +/- SD. All the experiments were performed in triplicate. No statistical difference vs. controls was present using unpaired t test with Welch's correction.
